# Living With and Managing Uncomplicated Urinary Tract Infection: Mixed Methods Analysis of Patient Insights From Social Media

**DOI:** 10.2196/58882

**Published:** 2025-03-11

**Authors:** Melissa L Kramer, Jose Medina Polo, Nishant Kumar, Aruni Mulgirigama, Amina Benkiran

**Affiliations:** 1 Live UTI Free Ltd Dublin Ireland; 2 University of Reading Reading United Kingdom; 3 Department of Urology, Hospital Universitario 12 de Octubre Madrid Spain; 4 Veeva Systems London United Kingdom; 5 GSK London United Kingdom

**Keywords:** acute cystitis, bladder infection, HCP interactions, urology, patient experience, patient insights, social media, uncomplicated urinary tract infection, urinary tract infection, urinary, women, quality of life, disease management, cystitis, healthcare professional, self-management, patient behavior, UTI

## Abstract

**Background:**

Uncomplicated urinary tract infections (uUTIs) affect more than half of women in their lifetime and can impact on quality of life. We analyzed social media posts discussing uUTIs to gather insights into the patient experience, including aspects of their disease management journey and associated opinions and concerns.

**Objective:**

This study aims to gather patient experience insights by analyzing social media posts that discussed uUTI.

**Methods:**

A search string (“urinary tract infection” [UTI] or “bladder infection” or “cystitis” or “UTI” not “interstitial cystitis”) was used to identify posts from public blogs and patient forums (June 2021 to June 2023). Posts were excluded if they were not written in English or discussed complicated UTI (posts that mentioned “pregnancy” or “pregnant” or “trimester” or “catheter” or “interstitial”). Posts were limited to publicly available sources and anonymized. The primary objective was to gather patient perspectives on key elements of the uUTI experience, including health care professional (HCP) interactions, diagnosis, treatment, and recurrence.

**Results:**

In total, more than 42,000 unique posts were identified (mostly from reddit.com; 29,506/42,265, 70%) and >3600 posts were analyzed. Posts were most commonly from users in the United States (6707/11,180, 60%), the United Kingdom (2261/11,180, 20%), Canada (509/11,180, 5%), Germany (356/11,180, 3%), or India (320/11,180, 3%). Six main themes were identified: symptom awareness and information seeking, HCP interactions, diagnosis and management challenges, management with antibiotics, self-management, and challenges with recurrent UTI. Most posts highlighted the importance of seeking professional medical advice, while some patients raised concerns regarding their HCP interactions and lack of shared decision-making. Patients searched for advice and guidance on the web prior to consulting an HCP, described their symptoms, and discussed lifestyle adjustments. Most patients tried self-management and shared their experiences with nonprescribed treatment options. There was general agreement among posts that antibiotics are necessary to cure UTIs and prevent associated complications.

**Conclusions:**

Social media posts provide valuable insight into the experiences and opinions of patients with uUTIs in Canada, Germany, India, the United Kingdom, and the United States. The insights from this study provide a more complete picture of patient behaviors and highlight the potential for HCP and patient education, as well as better communication through shared decision-making to improve care.

## Introduction

Urinary tract infections (UTIs) affect >50% of women in their lifetime [1,2], which can necessitate multiple health care professional (HCP) visits and antibiotic prescriptions, and have a substantial impact on quality of life [3,4]. Determinants of this quality-of-life impact include the capacity for symptoms to affect everyday activities (including sex, sleep, work, and exercise) and the emotional toll of disease management [5,6]. Frustration and helplessness are key examples of the emotions reported by patients with UTIs [5,7]. Most evaluations of patient perspectives of UTIs have focused on individuals recruited from clinical settings [8,9], but these data may be limited in terms of the information that patients are willing to share with researchers and the sample size may be small and not representative of the whole patient population. Other studies have gathered the views of patients with UTIs via web-based surveys [4,10-14].

Social media has become a part of everyday life for many people, with an estimated 4.89 billion users worldwide in 2023 [15]. This number is expected to reach 5.85 billion by 2027 [15]. Many patients use social media sites to source medical information prior to obtaining information in person [16]. These platforms thus represent a promising route by which we can learn about the concerns and experiences of patients without recruiting them from care settings or through research panels [17]. Analyzing patient discussions on social media may provide unique insights into the real-life experiences of patients beyond what can be learned using traditional research methodologies [17]. To our knowledge, only 2 studies have been published that present information about patients with UTI, extracted directly from web-based platforms (one of which was not UTI-specific) [16,18].

We analyzed social media posts discussing uncomplicated UTIs (uUTIs) to gather insights into the patient experience, including aspects of their disease management journey and associated opinions and concerns. uUTI (also known as “acute cystitis”) is a condition characterized by pyuria and a documented microbial pathogen on urine culture, often accompanied by local signs and symptoms such as lower abdominal discomfort and dysuria. uUTI is limited to nonpregnant females with no known relevant anatomical or functional abnormalities in the urinary tract and no comorbidities [19]. A quantitative analysis was also conducted to assess the proportion of posts discussing key uUTI therapies, home remedies, and HCP specialties. Refer to Multimedia Appendix 1 for “Patient Lay Summary” of this article.

## Methods

### Postidentification and Processing

**Figure 1:**
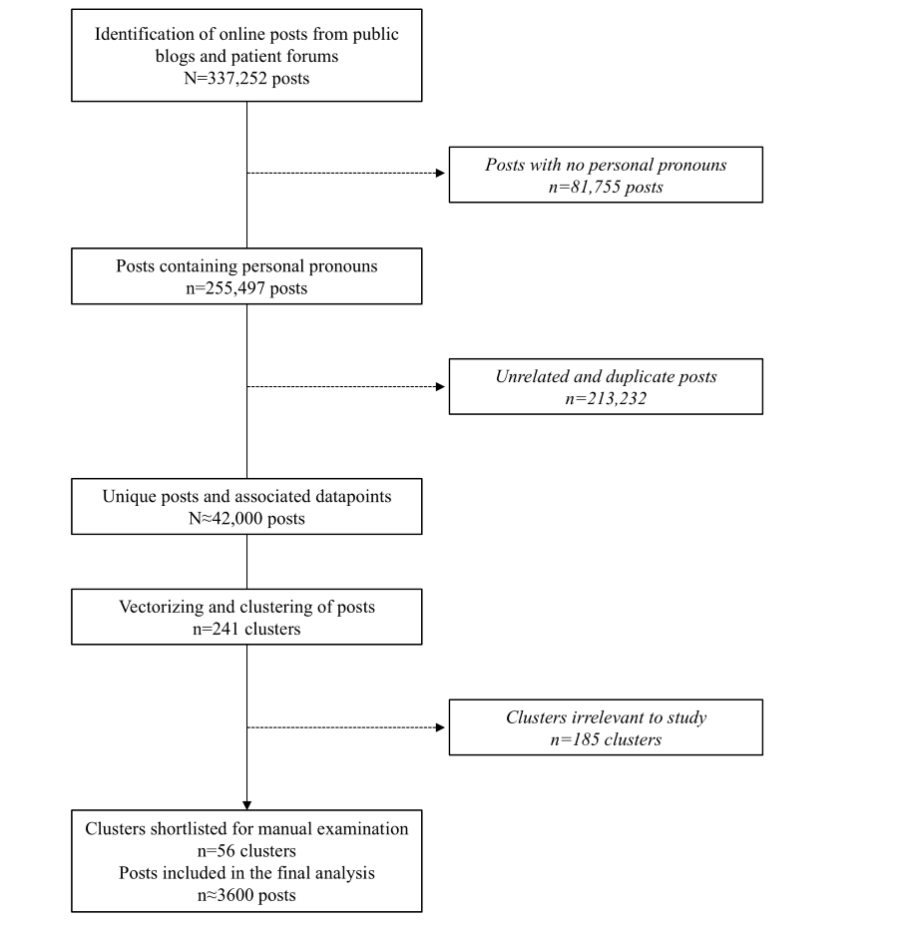
Data collection, extraction, and analysis.

A search string (“urinary tract infection” or “bladder infection” or “cystitis” or “UTI” but not “interstitial cystitis”) was used to identify online posts from Veeva Link data sets comprising multiple public blogs and patient forums from June 2021 to June 2023, as shown in Figure 1. Identified posts were subjected to natural language processing. Posts were excluded if they did not contain personal pronouns (to identify self-reports; examples of personal pronouns include I, me, my, mine, and myself), were not written in English, were deemed spam or advertisements (identified by manual inspection), discussed complicated UTI (identified as posts using the terms “pregnancy” or “pregnant” or “trimester” or “catheter” or “interstitial”), or were duplicates. Non–English-language sources, special characters, emojis, or website URLs included in the posts, duplicate posts, or posts that included terms associated with complicated UTIs were removed. Each post was assessed and clustered with other semantically similar posts.

The remaining posts were clustered into themes based on semantic similarities. Posts were embedded into a high-dimensional space using the all-mpnet-base-v2 transformer model and the dimensionality was reduced with Uniform Manifold Approximation and Projection [20] using the BERTopic framework [21] before clustering via HBDSCAN [22]. Model hyperparameters, such as minimum cluster size (n=10), were guided by intrinsic assessment of cluster quality such as coherence scores and extrinsic assessment of cluster quality through manual review. Topic interpretation for each cluster was aided by assessing the term frequency-inverse document frequency of keywords, and the GPT4 large language model was used to identify tangible themes for the clusters. The extracted keywords and GPT4 representations were assessed to determine which of the clusters warranted further manual examination to extract qualitative insights on uUTIs in terms of disease experiences, treatment experiences, prevention of UTIs, and experiences with HCPs. Clusters not relevant to uUTIs in these contexts were rejected.

### End Points and Analyses

The primary objective was to gather patient perspectives on key elements of the uUTI experience, including HCP interactions, diagnosis, treatment, and recurrence. Patient quotes are presented for each theme, and insights of interest are discussed alongside key datapoints (including website domains and countries of origin).

Quantitative data were also gathered to evaluate the frequency of posts discussing drugs and home remedies (including specific examples of each) and HCP specialties. This information is presented as a percentage of the total posts per country for countries with a sufficient sample size (the United States, the United Kingdom, Canada, Germany, and India). This analysis was performed prior to the launch of the Pharmacy First scheme (launched in the United Kingdom, January 2024) [23].

### Ethical Considerations

This research used secondary data only, was nonclinical and noninterventional, and was for patient experience insight purposes only, to support the current paucity of published data on patients’ perspectives with UTI. Institutional review board and research ethics committee approvals were not required nor sought because the research did not meet the requirements for needing ethical approval, per section 1.3 of the EphMRA guidelines [24]. The ethical principles established by the Declaration of Helsinki were respected. Consent to use data from the identified posts was considered implicit due to the published terms and conditions of each platform and the website privacy agreement with users (where relevant). Posts were limited to publicly available sources and anonymized to ensure that no personally identifiable information was included.

## Results

### Posts Included in the Analysis

A total of 337,252 posts were identified using the search string and 255,497 remained after filtering for those using personal pronouns (Figure 1). Removal of non-English posts, special characters, emojis, website URLs, posts deemed spam or advertisements, posts discussing complicated UTI, and duplicates gave ≈42,000 unique posts. The web-based source and geographic distribution of these posts is shown in Table 1. Most posts were taken from reddit.com (29,506/42,265, ~70%) and were most often from users in the United States (6707/11,180, 60%), the United Kingdom (2261/11,180, 20%), Canada (509/11,180, 5%), Germany (356/11,180, 3%), or India (320/11,180, 3%). After clustering, ≈3600 posts were identified for final analysis and manually examined to extract qualitative insights. Key takeaways were identified from each cluster, leading to the identification of 6 main themes from these posts. These themes were symptom awareness and information seeking, HCP interactions, diagnosis and management challenges, management with antibiotics, self-management, and challenges with recurrent UTI. Key patient quotes are included in Table 2.

**Table 1:** The web-based source and geographic distribution of posts.

Parameter			Web-based posts, n (%)
**Web-based source^a^**			
	reddit.com	29,506 (70)	
	tumblr.com	3973 (9)	
	whattoexpect.com	1869 (4)	
	healthunlocked.com	1453 (3)	
	babycenter.com	629 (1)	
	youtube.com	592 (1)	
	healingwell.com	271 (1)	
	healthcaremagic.com	264 (1)	
	nature.com	245 (1)	
	babycenter.ca	235 (1)	
	Other^b^	3228 (8)	
**Country of origin^c^**			
	United States	6707 (60)	
	United Kingdom	2261 (20)	
	Other^d^	1027 (9)	
	Canada	509 (5)	
	Germany	356 (3)	
	India	320 (3)	

^a^Total number of posts (N=42,265).

^b^Approximately 230 additional sites.

^c^Total number of posts where geographic information was available (N=11,180).

^d^Argentina, Australia, Austria, Bangladesh, Barbados, Belgium, Bosnia and Herzegovina, Brazil, Bulgaria, Chile, China, Costa Rica, Croatia, Czech Republic, Denmark, Estonia, Finland, France, Greece, Guam, Hong Kong S.A.R., Hungary, Iceland, Indonesia, Israel, Italy, Jamaica, Japan, Jordan, Kenya, Luxembourg, Macao SAR, Malaysia, Mexico, Morocco, Nepal, the Netherlands, New Zealand, Nigeria, North Macedonia, Norway, Pakistan, Panama, Paraguay, Peru, Philippines, Poland, Portugal, Puerto Rico, Qatar, Republic of Ireland, Romania, Russia, Saudi Arabia, Seychelles, Singapore, Sint Maarten, Slovakia, Slovenia, South Africa, South Korea, Spain, Sweden, Switzerland, Taiwan, Thailand, Trinidad and Tobago, Tunisia, Turkey, Ukraine, the United Arab Emirates, and Vietnam.

**Table 2:** Select patient quotes organized by theme.

Theme	Definition and verbatim quotes^a^
Symptom awareness and information seeking	This theme showcases a wide range of experiences and concerns related to UTI^b^ symptom awareness, prevention strategies, emotional impact, and peer support. The topic centers on UTI symptoms and guidance for managing suspected cases. “After exhausting all my options with doctors and gynaecologists, I am now turning to reddit and other women who may be able to help. I’ve been a sufferer of frequent UTIs for most of my adult life […] Every time I have a period, or am about to have a period, I develop a UTI, there seems to be no rhyme or reason on whether it’ll happen just before or just after, but without fail, I’ll always get one.” “It sounds like you have a urinary tract infection. It’s pretty easy to treat but you do need to go to a doctor or urgent care […] to be sure.” “So my friend recently got a bladder infection (we think) she’s a bit nervous about going to the doctor for various reasons, so we’re looking for natural suggestions that might work […] She doesn’t have any serious symptoms yet, just the general discomfort while peeing and the constant urge to pee and unintentional leaking but we just don’t want it to escalate and want some tips before going to a doctor.” “You can try taking cranberry tablets they work for some people. Also drink plenty [of] water after and try to pee again if you can! If the problem persists you can get an antibiotic to take each time you have sex from the doctor.”
HCP^c^ interactions	This theme predominantly revolves around the frustration individuals with UTI symptoms feel when seeking treatment from HCPs. “Doctor has suggested [low dose antibiotic] as I feel like I have cystitis symptoms all the time for last 2 months, got to wait 6 months to see urologist to see what problem is. My GP^d^ said they don’t have the answers, a lot of it is trial and error. I know myself better than them so I’m always looking and trying different things.” “I went to the Dr because I suspected a bladder infection. Gave all my symptoms. […] Only after pushing did he condescendingly ask if I’d feel better if I provided a urine sample. I said yes. He couldn’t look me in the eyes when he gave me a prescription for you [guessed] it, a bladder infection. I’ve learned that if I’m dealing with my [health] to push back if I feel I am not listened to.” “It took visits to my general physician (who, in less words, told me ‘Just stop having sex’ LOL), my gynecologist, and then a urologist she recommended, before I got relief. I’m gonna tell you, have your girlfriend see a FEMALE urologist, if you can. Male urologists unfortunately don’t always keep up with female urinary research (it’s extremely behind male urinary research, too) and having someone who could relate to me and understood my pain as a female/AFAB^e^ was HUGE.”
Diagnosis and management challenges	This theme primarily centers around obstacles that individuals experiencing UTI symptoms encounter when seeking an accurate diagnosis from HCPs. “OMG!!! I knew I had a UTI and the [….] doctor kept refusing to test saying it was probably an STD^f^. It was not an STD and when the results for the UTI came back it showed I had a staph infection in my bladder.” “The first time I was quite happy to go through the rigamarole of giving a urine sample and getting antibiotics. After a few episodes of this happening, the antibiotics started playing with my gut and causing issues there […] four years in I have a system that has worked well enough for me.” “I had a WOMAN doctor tell me the blood coming from an intense bladder infection was… and I quote, ‘Probably a miscarriage.’ I had been going through a divorce for months and hadn’t had any physical contact, let alone the [contraceptive] I was on. She didn’t even ask. The bias against women’s health is incredibly ingrained in the entire system.” “He put me on Flagyl, which immediately gave me a yeast infection, put me on another med, got the UTI back. He put me on Flagyl again, and another yeast infection. Apparently it’s a side effect. This cycle went on about 8 times, and every time, I told him the meds weren’t working and giving me more problems. But he’s the doctor and knows best, right? Weeks later, I’m in ICU^g^ literally clinging to life with sepsis, because of an ‘undiagnosed UTI’.” “Doing a more in-depth test cannot hurt […] it’s sad all they do is dip stick, culture, cipro […] I would have never found my staph infection in my bladder had I not tested further using the advanced next generation sequencing DNA test.”
Management with antibiotics	This topic focuses on physician-led treatment approaches to managing UTIs, including personal experiences with the antibiotic use, their pros and cons, and potential side effects. “I had, maybe still have, a super resistant [*Pseudomonas*] UTI. I’m still having occasional burning in the bladder area and occasional back pain a week after stopping [antibiotic] (really bad side effects, had to stop after 4 days, but doctors said I took enough of it) […] my urine is clear now despite my symptoms. They still sent it for culture and I’m waiting on that to come back. I am so distressed from this, it’s taking a huge toll on me as it’s been going on for over two weeks back and forth with doctors. Also, especially after reading about [*Pseudomonas*], I feel like I won’t be able to get rid of it and who knows what will happen to me.” “I had this same problem for years. At one point I had 5 UTIs in 4 months. Every time it would be after sex with my husband. The doctor cultured it but could never identify what specifically was causing the UTI. Finally, when I saw my gynecologist she gave me a prescription for [antibiotic]. I take one pill right after sex. I’ve been UTI free for the last 15 years.” “I ask because the [antibiotic] is causing me a lot of pain in the left leg and I’m scared to continue it […] I had three of the six I was told to take before I stopped because I couldn’t sleep or walk well from the pain (which slowly died down over the day I was off them) but now I’ve taken a fourth because the UTI scares me.” “If [antibiotic] hasn’t worked after the 1st course we generally want to switch to something else rather than bombard the body with long-term antibiotics. That’s just a recipe for an ongoing yeast infection, lowered gut health and a massive case of [*Clostridium difficile*] diarrhea.”
Self-management	This topic focuses on self-management approaches to treating UTIs, including recommendations regarding different approaches to managing UTIs such as home remedies. “UTIs are more common in women than men. If you are male definitely go to the doctor […] If you are female, you are supposed to go to the doctor, but honestly most just drink water and low sugar cranberry juice until they’re fine because they’re so common. If you get a fever, definitely go to the doctor.” “Stop just using cranberry juice every time you have a UTI!! So many [women] go through so much unnecessary pain and worsen their UTIs just because they think drowning themselves in cranberry juice will help. Cranberry juice/cranberry supplements can HELP PREVENT UTIs, once you have one, your best friend is going to be antibiotics, pain killers, and WATER to flush out bacteria.” “Just wondering if anyone has tried D-Mannose for recurrent UTI? I have a prescription for [antibiotic] to take after intercourse or when I feel a UTI coming on but I take it so often that I’m worried about antibiotic resistance. I use Azo a lot but it stains... I’m trying to find something to help as a preventative.” “I’ve had great success with using D-Mannose. Since I had a UTI in [February] I was left with chronic low-level symptoms that didn’t go away—awareness, bladder burning, frequency. My culture came back negative but after reading about embedded infections I began to suspect that it was an infection that was just going undetected. I use powder D-Mannose (more potent than capsules). After about a week of high dosing (4× a day) my symptoms were greatly reduced, almost completely.” “I used to get UTIs, and having to go to the doctor placing me on different kinds of antibiotics. Some worked, some didn’t so I wound up having to go back to the doctor which winds up to be very expensive [...] I came across a video on YouTube where this girl [...] said she was taking a probiotic plus something for UTIs. She was taking a product called D-Mannose. I ordered it from Amazon, and I have to say that it has worked for me.”
Challenges with recurrent UTI	This topic primarily centers on individuals’ experiences with recurrent UTIs. Discussions focus on personal experiences, physical and mental burden, and methods used for both treatment and prevention. “I don’t know if my fourth and most recent [antibiotic] is working against this, and my doctor won’t be in for the next five days. I’m so tired of this! I know that rUTIs^h^ is a thing, but to have these literally back to back? No day to breathe without wondering if this will be when I go septic and die? I almost wish that this will kill me at this point.” “I’ve been to urologists and a [urogynaecologist] in addition to my [gynaecologist]. No one can give me answers. My cystoscopy was fine and after that, the [urogynaecologist] said to just take dmannose daily, drink 3-4 liters of water a day, and good luck. I refuse to believe this will be my life now [...] There HAS to be some resolve.” “I’ve struggled with recurrent UTIs for about 15 years and tried every single supplement and herb I could find info about. I often react very poorly to antibiotics (ranging from allergic reactions to intestinal yeast infection, oral thrush, diarrhea for weeks, flu-like symptoms, I [hate] taking antibiotics) so I avoid them whenever possible, I’ll do anything to cure UTIs at home before resorting to antibiotics.” “LOOKING FOR ADVICE! I suffer from recurring urinary tract infections. When I go to the doctor, they just put me on antibiotics for a few days and the infection usually goes away […] I don’t want to be on antibiotics that often and ruin my microbiome. However, I don’t want to risk permanent damage to my kidneys by not treating the UTIs properly. I am looking for tips on how to better prevent them moving forward.”

^a^Quotes have not been edited, except for omission of text where deemed appropriate (indicated by [...]) or correction of spelling or grammar, removal of drug names, and definition of non–standard abbreviations for clarity (indicated by corrected or new text).

^b^UTI: urinary tract infection.

^c^HCP: health care professional.

^d^GP: general practitioner.

^e^AFAB: assigned female at birth.

^f^STD: sexually transmitted disease.

^g^ICU: intensive care unit.

^h^rUTIs: recurrent urinary tract infections.

### Symptom Awareness and Information Seeking

Many posts were from individuals seeking guidance or recommendations for when faced with UTI symptoms and methods for managing symptoms at home. Posts shared personal experiences (including physical and mental symptoms, HCP and patient experience, and impact on daily life and activities) with UTIs and expressed support for fellow individuals with UTIs. Patients value the ability to form virtual networks and communities with others facing the same challenges. Some posts provided recommendations for pain relief and symptom management strategies. One post stated, “So many [women] go through so much unnecessary pain and worsen their UTIs just because they think drowning themselves in cranberry juice will help. [...] Once you have one, your best friend is going to be antibiotics, pain killers, and WATER to flush out bacteria.” 

Some posts highlighted a tendency for patients to seek guidance on the web prior to consulting an HCP. Some patients do this because they feel uncomfortable approaching an HCP or because they prefer to determine the severity of their symptoms on the web before seeking professional help. One post highlighted this hesitancy: “My friend recently got a bladder infection (we think) she’s a bit nervous about going to the doctor for various reasons [...] She doesn’t have any serious symptoms yet [...] but we just don’t want it to escalate and want some tips before going to a doctor.”

Notably, many posts stressed the importance of seeking professional medical help when confronted with UTI symptoms. One post said, “It’s pretty easy to treat but you do need to go to a doctor or urgent care [...] to be sure.”

### HCP Interactions

Posts frequently discussed difficulties obtaining a medical appointment. Some patients highlighted long waiting times and others were advised to visit the emergency department, which was often deemed an inappropriate course of action. One post stated, “Doctor has suggested [low dose antibiotic] as I feel like I have cystitis symptoms all the time for last 2 months, got to wait 6 months to see urologist to see what problem is.” Patients who had attended appointments often discussed the tendency for HCPs—particularly males—to be dismissive of the impact of their UTI symptoms. Some patients equated this to trivializing their pain and discomfort. In contrast, female HCPs were reported to be more empathetic and demonstrated a deeper comprehension of the specific requirements of female patients. One post read, “Have your girlfriend see a FEMALE urologist, if you can. Male urologists unfortunately don’t always keep up with female urinary research.”

The medical approaches relayed in the posts demonstrated a lack of shared decision-making. Many HCPs prescribed treatments without engaging in comprehensive discussions with patients. This practice led patients to feel frustrated, particularly when treatment was ineffective or had unfavorable side effects. Some posts offered advice for navigating HCP appointments, including specific diagnostic evaluations or treatments to advocate for, and which to avoid. The importance of advocating for personal preferences was emphasized. One patient said, “I’ve learned that if I’m dealing with my [health] to push back if I feel I am not listened to.”

### Diagnosis and Management Challenges

Many patients commented on complex diagnostic processes or a lack of diagnostic workup before receiving a prescription. The restricted sensitivity of urine culture tests was discussed (particularly for less prevalent strains or infections with minimal bacterial presence) and some posts advocated for more sensitive assessments such as the MicroGenDX diagnostics test (which combines quantitative polymerase chain reaction with 16s/18s next-generation sequencing). One post read, “Doing a more in-depth test cannot hurt [...] it’s sad all they do is dip stick, culture, cipro [...] I would have never found my staph infection in my bladder had I not tested further using the advanced next generation sequencing DNA test.” Patients also reported instances of misdiagnosis while experiencing UTI symptoms, including diagnoses of period cramps or sexually transmitted infections. One patient said, “I knew I had a UTI and the [...] doctor kept refusing to test saying it was probably [a sexually transmitted disease (STD)]. It was not an STD and when the results for the UTI came back it showed I had a staph infection in my bladder.” Misdiagnosis led to a loss of trust in HCPs.

Posts also discussed the provision of inadequate treatment by medical professionals. One read, “I went to the [doctor] because I suspected a bladder infection. Gave all my symptoms. [...] Only after pushing did he condescendingly ask if I’d feel better if I provided a urine sample. I said yes. He couldn’t look me in the eyes when he gave me a prescription for you [guessed] it, a bladder infection.”

Some patients felt that treatment decisions should be informed by comprehensive diagnostic evaluations. Instances of antibiotic prescribing without consideration of the specific bacteria present were highlighted. These situations often led to ineffective treatment that necessitated multiple rounds of therapy.

### Management With Antibiotics

There was consensus among posts that antibiotics are necessary to cure UTIs and prevent associated complications. Nitrofurantoin was the most frequently mentioned antibiotic, followed by ciprofloxacin, but many patients said that they were frustrated with medical interventions as they continue to experience UTIs despite good adherence. Few posts described situations where HCPs conducted microbiological cultures and sensitivity testing to identify the most appropriate antibiotic for their UTI. Some patients expressed a preference for more convenient doses, with 1 patient explaining that this was due to the need to “choke down pills [twice a day]” on [antibiotic].

Side effects of antibiotic therapy were also discussed and concerns regarding antibiotic resistance led some patients to seek alternative therapy. One patient asked, “Just wondering if anyone has tried D-Mannose for recurrent UTI? I have a prescription for [antibiotic] [...] but I take it so often that I’m worried about antibiotic resistance. [...] I’m trying to find something to help as a preventative*.*”

Patients who chose not to take certain classes of antibiotics reported opting for home remedies or other antibiotics instead. Discussed side effects included nausea, potential lung toxicity, and an increased risk of yeast infections. A small number of patients discussed HCP warnings about potential tendon-related side effects when taking certain antibiotics. Patients also discussed concerns regarding the associated black box warning and some reported experiencing leg and back pain while on treatment. One post said, “It has 3 black box warnings and has caused healthy people horrible side effects.” Another said, “[Antibiotic] is causing me a lot of pain in the left leg and I’m scared to continue it.” Within the posts, there were no references to shared decision-making when prescribing antibiotics.

### Self-Management

Over-the-counter and home remedies for UTI were the most common topics among posts but opinions on them varied. Some patients preferred self-management as it helped them feel in control of their treatment. These patients may have also felt frustrated with the care they received or with having to return to the doctor multiple times. One patient said, “I used to get UTIs, and having to go to the doctor placing me on different kinds of antibiotics. Some worked, some didn’t so I wound up having to go back to the doctor which winds up to be very expensive.”

Patients with a positive view of home remedies shared their experiences with nonprescribed treatment options, including hydration, D-mannose, cranberry, probiotics, and herbal supplements (uva ursi). Opinions on cranberry juice and supplements were more divided; this seemed to be the case among both patients and HCPs. One post about cranberry products read, “If you are female, you are supposed to go to the doctor, but honestly most just drink water and low sugar cranberry juice.” Another said, “Stop just using cranberry juice every time you have a UTI!! So many [women] go through so much unnecessary pain and worsen their UTIs just because they think drowning themselves in cranberry juice will help.”

### Challenges With Recurrent UTI

Posts about recurrent UTI consistently highlighted the burden of persistent and incapacitating symptoms and their association with feelings of anxiety and helplessness. One post read, “No day to breathe without wondering if this will be when I go septic and die[.] I almost wish that this will kill me at this point.” Factors contributing to the emotional toll of recurrent UTI included a perceived loss of bodily control and a negative impact on intimate relationships. Some posts discussed the use of long-term, low-dose antibiotics to prevent recurrence but lacking effectiveness and side effects were also mentioned. Many patients turned to over-the-counter remedies such as D-mannose and cranberry products to prevent recurrence. Success with these approaches was limited. One patient said, “I’ve struggled with recurrent UTIs for about 15 years and tried every single supplement and herb I could find info about. I often react very poorly to antibiotics [...] I’ll do anything to cure UTIs at home before resorting to antibiotics.” Other patients reported lifestyle changes (such as reducing caffeine and alcohol intake and stringent hygiene routines) to combat recurrent UTI and some reported positive outcomes of these changes.

### Quantitative Outcomes: Antibiotics, Home Remedies, and HCP Specialties

**Figure 2:**
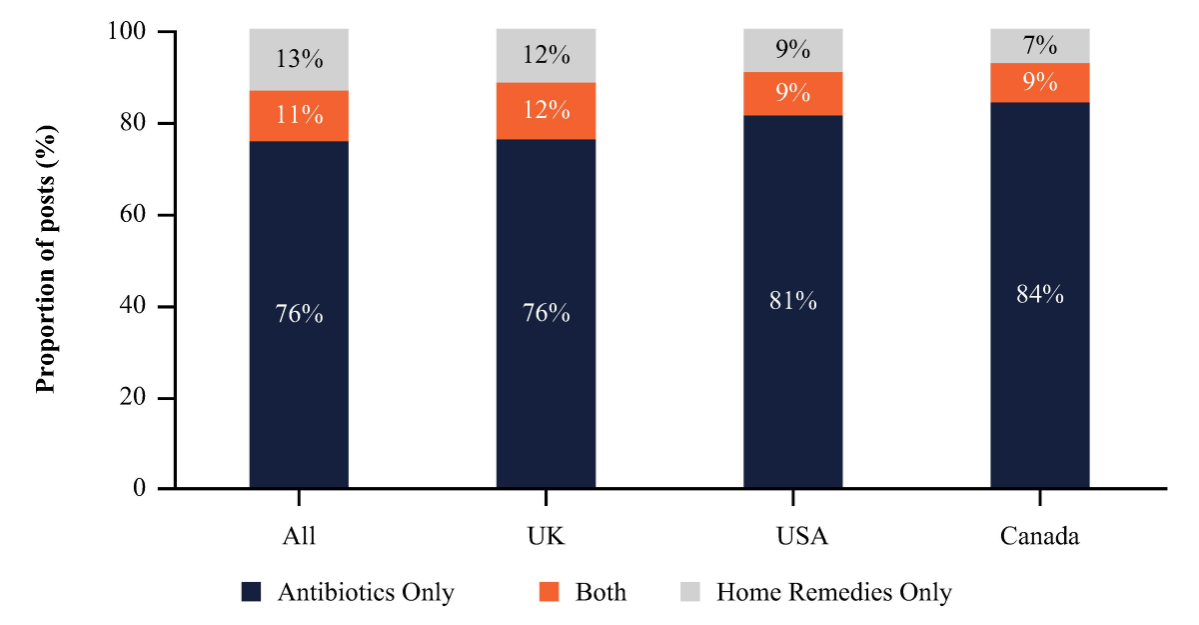
Proportions of posts mentioning antibiotics only, home remedies only, or both antibiotics and home remedies. Home remedies included D-mannose, cranberry products, probiotics, and herbal supplements.

All countries (Canada, Germany, India, the United Kingdom, and the United States) were included in the analysis of 9619 posts mentioning antibiotics, home remedies, and HCP specialists. Most posts (7270/9619, 76% of those included in the quantitative analysis) discussed antibiotics only, 13% (1290/9619) mentioned home remedies only, and 11% (1059/9619) mentioned both antibiotics and home remedies, as shown in Figure 2. The proportions of posts mentioning antibiotics and home remedies were similar across Canada, the United Kingdom, and the United States, but specific drugs (including antibiotics and pain relief) were discussed less frequently in the posts than home remedies. Cranberry and D-mannose were the most discussed home remedies.

**Figure 3:**
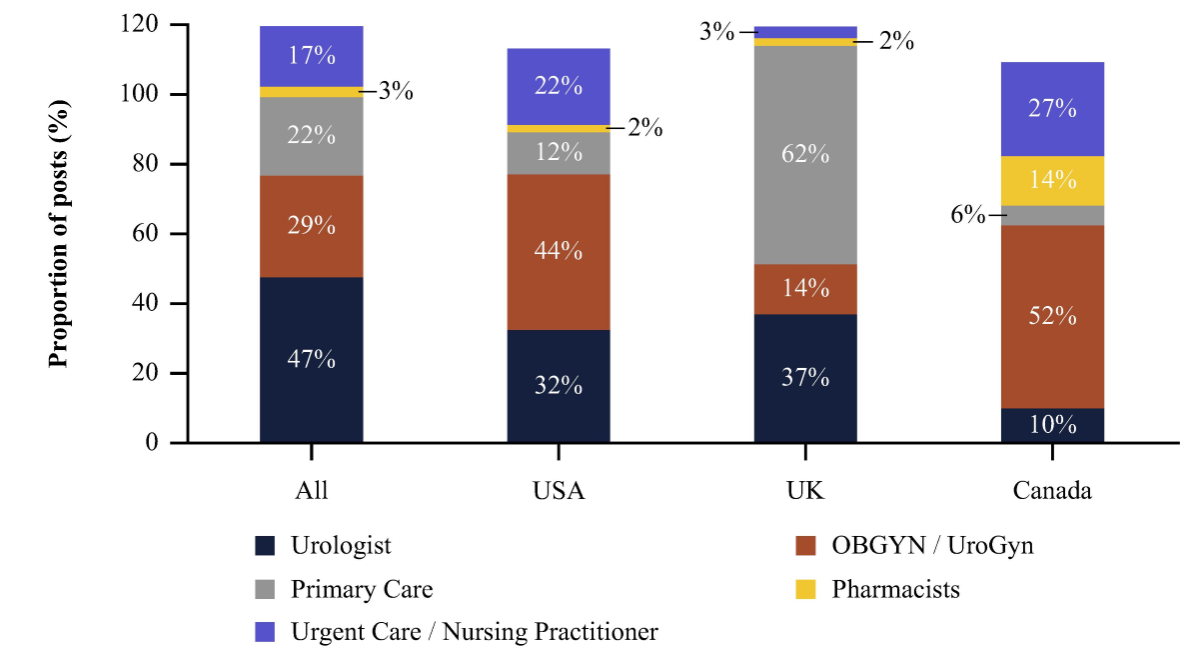
Proportions of posts mentioning health care professional specialties. OBGYN: obstetrician/gynecologist. UroGyn: urogynecologist.

Figure 3 shows that, among posts mentioning HCP specialties, specialist HCPs (urologists, obstetricians or gynecologists, and urogynecologists) were mentioned more frequently in posts from the United States (670/875, 77%) and Canada (44/71, 62%) than from the United Kingdom (329/646, 51%). Only 3% (21/646) of posts from the United Kingdom discussed nurse practitioners or urgent care versus 27% (19/71) in Canada and 22% (190/875) in the United States. Mentions of pharmacists were also much more frequent in Canada (10/71, 14%) than in the United Kingdom (14/646, 2%) or the United States (18/875, 2%).

## Discussion

### Principal Findings

This retrospective social media study highlighted the strength of the web-based uUTI community, in which patients provide one another with emotional support and recommendations based on personal experience. Topics and behaviors observed in this study were somewhat aligned with those noted in the only preexisting, large-scale social media analysis of uUTI, including information-seeking, antibiotic treatment, and alternative therapy [18]. In addition to these topics, our study raised some further themes, including discussions about interactions with HCPs, as also observed in the study by Flower et al [8]. These insights have important implications for the patient journey and challenges associated with recurrence and should supplement existing data to provide a more holistic view of patient experiences and opinions of uUTI.

Patients in this study tended to search for advice on the web before consulting an HCP, and most posts stressed the importance of seeking professional help. Nevertheless, not all patients obtained medical appointments easily or were satisfied with their HCP interactions once an appointment was secured. Patients also reported that some HCPs lacked empathy and knowledge of the potential emotional, mental, and physical impact of uUTIs on patients. Others were frustrated with treatments prescribed following inadequate discussions. These sentiments are reflected in the findings of previous qualitative studies of UTI [7,11]. In one such study, 35% of 466 survey respondents highlighted dismissive or unempathetic HCP behavior and many described difficulties accessing care [11]. Another study noted miscommunication between patients and HCPs as a barrier to appropriate prescribing [7]. Effective HCP communication regarding the advantages and drawbacks of certain therapies is essential to foster meaningful shared decision-making. Some posts also highlighted female HCPs as more empathetic than males. This is important because HCP empathy can improve patient satisfaction, increase adherence, and enhance outcomes [25,26]. Patients in a prior clinical study based in an obstetrics and gynecology department reported preferences for female HCPs because of their empathy and competence [27].

Most patients in this analysis also discussed the importance of antibiotics to cure their UTI. However, some patients had concerns about antibiotic use related to persistent symptoms and to the knowledge that repeated use of antibiotics may lead to antibiotic resistance, prompting them to seek alternative therapy. Hesitance in using antibiotics has also been associated with concerns that antibiotics may not be effective in the future; some women would rather understand how and why they got the UTI and how to prevent it in the future [7]. These concerns could be alleviated by ensuring that patients receive appropriate information on prevention and potential antimicrobial resistance, are prescribed the most appropriate treatment (especially those at risk of developing symptoms due to antibiotic-resistant bacteria), and feel empowered to engage in shared decision-making during treatment discussions with HCPs. Risk factors associated with antibiotic resistance in UTI include older age, which could be due to previous antibiotic exposure [28], and recurrent UTI [29].

Fears of antibiotic resistance in UTI may be linked to the common misconception that antibiotic resistance is a quality intrinsic to patients, rather than to the bacteria causing an infection [7,18]. This phenomenon may explain the prior observation that women with UTI are generally willing to accept treatment options with a longer time to resolution or a higher risk of side effects to avoid resistance [12]. Combined with factors such as hesitancy to see an HCP and frustration with care, fears of resistance may also contribute to the prevalence of posts about home remedies for UTI. This was the most common topic in our study. Many posts highlighted experiences with cranberry products and D-mannose, which are supported by mixed (positive and negative) and low-level clinical trial evidence, respectively [30]. In fact, a prior social media study identified that 53% of web-based recommendations had some supporting evidence (including cranberry products and D-mannose) but 43% were not supported by science. These unsupported recommendations included hygiene routines and dietary modifications such as avoiding alcohol and caffeine [16]. Web-based recommendations should be taken with care as the people advising may not be medical professionals. According to the European Association of Urology Guidelines on Urological Infections, clinicians may recommend cranberry products and D-mannose to reduce recurrent UTI episodes in patients, providing they inform them of the weak evidence base supporting this [31]. Overall, there are few studies and a lack of robust data investigating these recommendations, and this is an ongoing challenge in UTI research. It is interesting that, despite lacking placebo-controlled data and evidence for these approaches, a substantial number of posts in our study reported success when using them to prevent recurrent UTI; however, the true efficacy of these measures using placebo-controlled trials is undetermined.

The quantitative results obtained were unsurprising and reflect geographical variations in access to health and everyday practice behaviors. For example, while patients in Canada and the United States can access specialists without needing a referral, in the United Kingdom, National Health Service specialist appointments require a referral from a general practitioner. This is reflected in the high proportion of patients mentioning primary care (402/646, 62%) in UK posts. Similarly, pharmacists were mentioned more frequently in Canada because of the expanded scope of their role, which includes the capacity to prescribe antibiotics for UTI, as opposed to countries where it does not.

Patients from this analysis tended to seek information via social media before approaching an HCP, which underscores a potentially important opportunity for information outreach. Patients seeking information related to health online is becoming increasingly popular [32]; this need is emphasized by the fact that many patients lack preexisting knowledge regarding the cause, diagnosis, and treatment of UTIs [33]. Currently, however, many posts on social media sites are from sources that are not peer-reviewed and may contain misinformation [16,34]. Literature has highlighted that poor health literacy may affect the usefulness of social media interventions, which may result in health inequity [35]. One analysis on the video-sharing platform TikTok found that 75% (224/300) of identified videos about antibiotics were from non-HCPs, but HCP videos were significantly more relevant, significantly less biased, and significantly more likely to have an educational focus [34]. This highlights the need for accurate and successful advocacy for patients. An emphasis on creating HCP-led educational content for these platforms may help patients who seek health information via social media to identify the correct care sooner. Educating patients in this way could improve their communications with HCPs and drive shared decision-making. However, it is crucial to note that some HCPs may lack up-to-date knowledge on the testing and treatment approaches for uUTI, especially recurrent UTI, leading to patients feeling misunderstood [14]. It is important that both HCPs and patients are educated on uUTI and shared decision-making. This approach might also limit the potential for negative patient-HCP interactions driven by the acquisition of suboptimal information from non-HCPs on the web [36]. One notable obstacle may be the lack of social media use among HCPs: a 2021 analysis of 650 HCPs at 10 top American hospitals showed that more than 70% had at least 1 social media profile but among these, almost 90% had not posted in the prior month [37].

### Limitations

This study had some limitations that must be considered when interpreting the outcomes. Demographic data were not collected and most content was posted by users in Canada, the United Kingdom, and the United States (mainly due to the inclusion or exclusion criteria for the posts); views from some patient groups and from other countries may be underrepresented. One patient group that may be particularly underrepresented is older patients, who may use social media less than their younger counterparts. It is also possible that patients posting on social media may have had more troublesome UTI experiences (such as more impactful symptoms or a worse treatment journey) than those who chose not to post on social media. While every effort was made to limit results to those associated with uUTI, some cases of complicated UTI or comorbidities may have been included, which could have an impact on the analysis. Posts from closed forums were not included in the analysis. Valentine-King et al [11] reported that surveys may be distributed using an international internet platform, reaching a larger group of patients, not limited to a particular practice, health network, or country. This may increase the variety of responses and experiences [11], although participation could still be limited for older patients or those with no or limited access to technology [38].

### Conclusions

This analysis of more than 42,000 social media posts provides valuable insight into the experiences and opinions of patients with uUTI in Canada, Germany, India, the United Kingdom, and the United States. Many patients sought information on the web before seeking professional medical care, which emphasizes the importance of peer support in the UTI patient journey and the value of accessible web-based resources for patient education. Women reported difficulties accessing care and inadequate interactions with HCPs, characterized by dismissive behavior and lacking diagnostic evaluations. Although the importance of antibiotics was widely appreciated, many patients noted hesitance toward them or dissatisfaction with their efficacy and side effects. Patients highlighted fears and concerns about risks of antimicrobial resistance which, if proactively discussed by HCPs with patients, could further improve patient-HCP interactions. Alternative treatments were discussed frequently but not all were supported by scientific evidence. Overall, the insights gleaned in this study provide a more complete picture of patient behaviors and highlight the potential for HCP and patient education in addition to improved communication with HCPs to improve care.

## Appendix

Multimedia Appendix 1 Patient lay summary.

## Abbreviations

HCP: health care professional
UTI: urinary tract infection
uUTI: uncomplicated urinary tract infection
