# Supplementary material for: Living With and Managing Uncomplicated Urinary Tract Infection: Mixed Methods Analysis of Patient Insights From Social Media
Source: J Med Internet Res. 2025 Mar 11;27:e58882. doi: 10.2196/58882 (PMC11937705; doi:10.2196/58882)
Supplement: Multimedia Appendix 1 [file jmir_v27i1e58882_app1.docx]

### Patient Lay Summary

More than half of the women will have a bladder infection (also known as “acute cystitis” or an “uncomplicated urinary tract infection [uUTI])” in their lifetime. We conducted this study to better understand how bladder infections affect women emotionally and physically, including their perception of interactions with medical professionals. To do this, we collated social media posts made by women about bladder infections and looked at the information they discussed. In this study, we found that most women with a bladder infection looked for advice on the web before they spoke with their doctor. We also found that there is a need for better communication between women with bladder infections and their doctors to improve patient-doctor interactions and patient outcomes. Our study provides valuable information from the patient’s perspective.
